# Supplementary material for: Inhibition of the Lysophosphatidylinositol Transporter ABCC1 Reduces Prostate Cancer Cell Growth and Sensitizes to Chemotherapy
Source: Cancers (Basel). 2020 Jul 23;12(8):2022. doi: 10.3390/cancers12082022 (PMC7465469; doi:10.3390/cancers12082022)
Supplement: Supplementary file 1 [file cancers-12-02022-s001.pdf]

Supplementary Materials: Inhibition of the Lysophosphatidylinositol Transporter ABCC1 Reduces Prostate Cancer Cell Growth and Sensitizes to Chemotherapy

Aikaterini Emmanouilidi, Ilaria Casari, Begum Gokcen Akkaya, Tania Maffucci, Luc Furic, Federica Guffanti, Massimo Broggini, Xi Chen, Yulia Y. Maxuitenko, Adam B. Keeton, Gary A. Piazza, Kenneth J. Linton and Marco Falasca

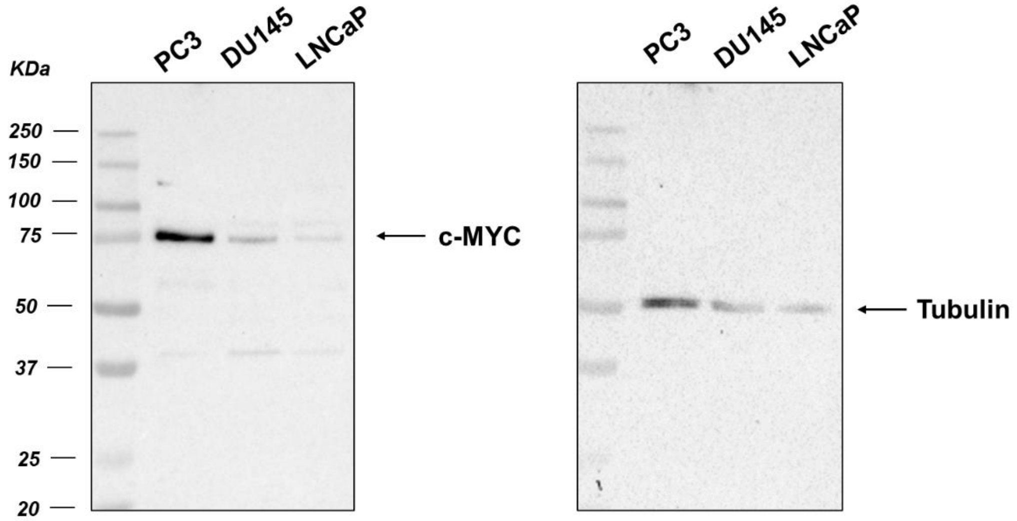

Figure S1. Expression of c-MYC in prostate cancer cells. Lysates from the indicated prostate cancer cell lines were analysed by Western blotting using an anti-c-MYC antibody. Membranes were stripped and re-incubated with anti-Tubulin to confirm equal loading.

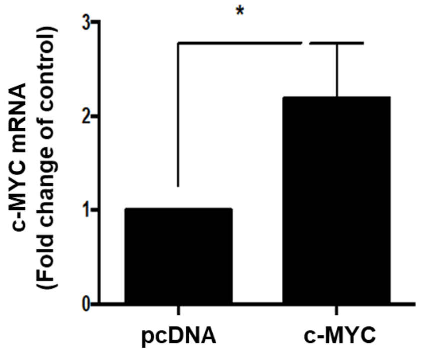

Figure S2. Efficient overexpression of c-MYC. PNT2 cells were transfected with a plasmid encoding c-MYC or the corresponding empty vector (pCDNA). c-MYC mRNA levels were determined by RT-qPCR. Data are expressed as fold change of mRNA levels in cells transfected with empty vector (control) and are means + SD of $n = 3$ experiments. * $p < 0.05$.

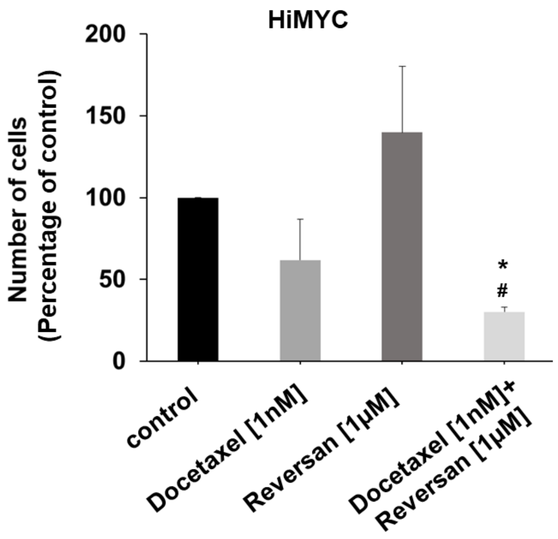

Figure S3. Effect of Docetaxel and Reversan on HiMYC cells. HiMYC cells were treated with the indicated concentrations of Docetaxel or Reversan alone or in combination. Control cells were treated with vehicle (DMSO). Number of cells was assessed after 72 h by cell counting. Data are expressed as percentage of results from control cells and are means + SEM from $n = 3$ independent experiments. * $p < 0.05$ vs Docetaxel; # $p < 0.05$ vs Reversan.

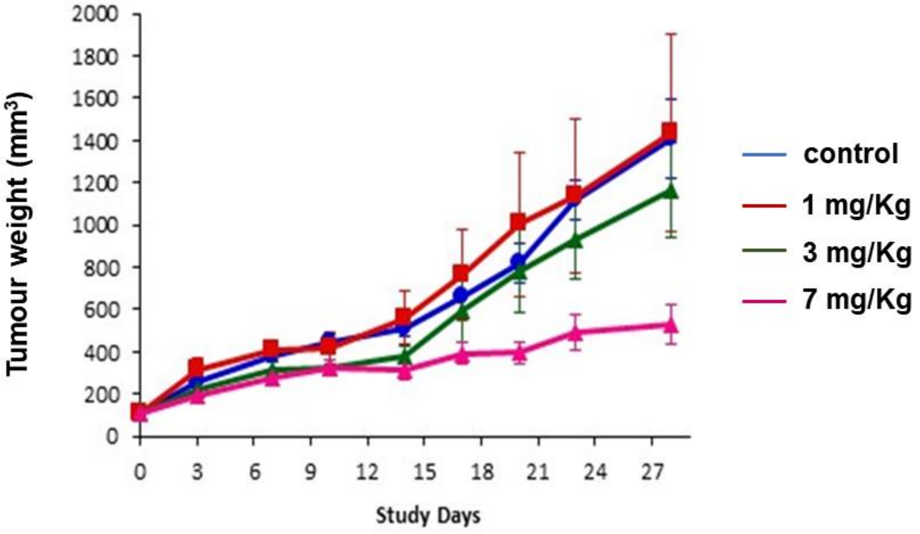

Figure S4. Effect of different concentrations of Docetaxel on a xenograft prostate cancer cell model. PC3 cells were inoculated subcutaneously in male athymic nude-Foxn1^{nu} mice and treated with the indicated concentrations of Docetaxel or vehicle (control). Data show tumour weight at the indicated days from start of treatment.

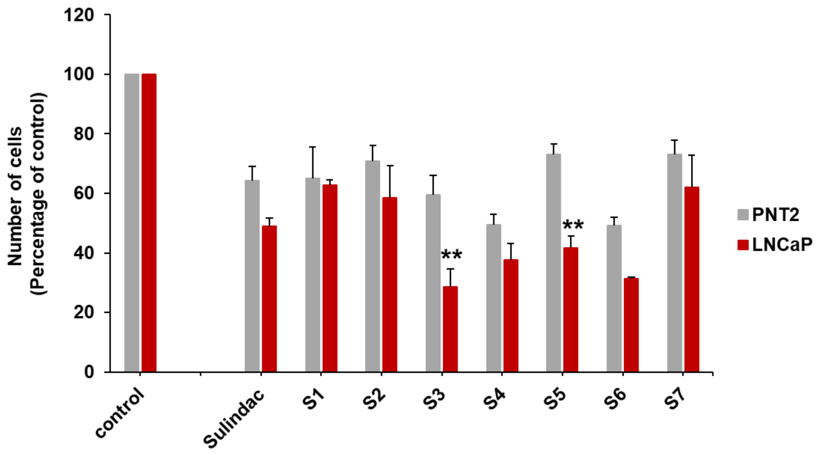

Figure S5. Effect of Sulindac and its derivatives. PNT2 and LNCaP cells were treated with Sulindac and a panel of its derivatives (10 μM). Control cells were treated with vehicle, DMSO alone. Number of cells was assessed after 72 h by cell counting. Data are expressed as percentage of number of control cells and are means + SEM from $n = 3$ independent experiments. ** $p < 0.01$ vs corresponding PNT2 values.

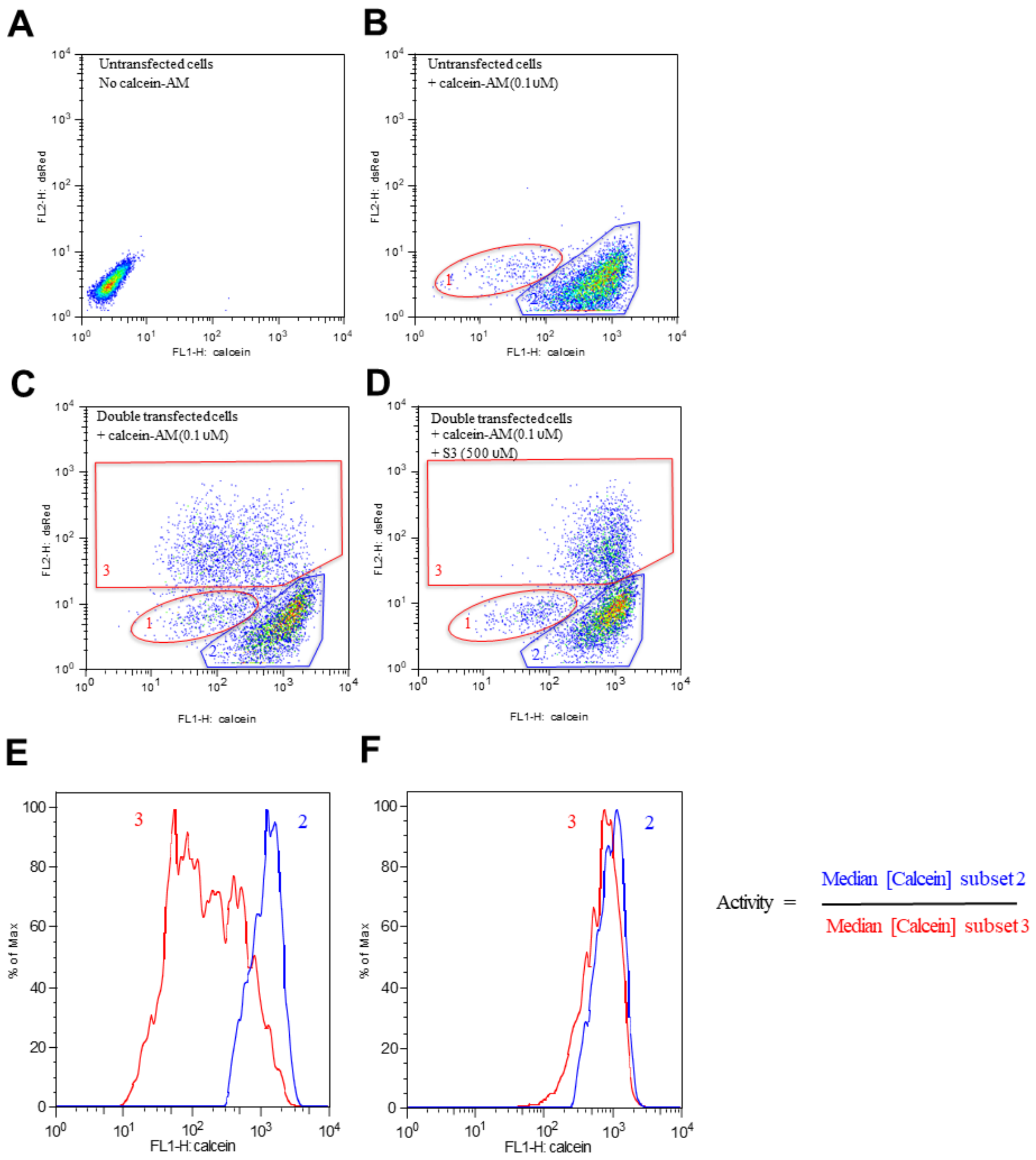

Figure S6. Flow cytometric assay of ABCC1 function and inhibition. Example of the raw data from ABCC1 transport inhibition data. 10,000 live, untransfected cells of normal size and granularity in the absence (panel **A**) or presence (panel **B**) of Calcein-AM. Calcein-AM is a hydrophobic compound, which readily penetrates the plasma membrane of cells. In the cytosol, esterases hydrolyse the acetomethoxy (AM) group from this colourless dye to release the green-fluorescent Calcein (panel **B**). About 3 % of our HEK293T cells fail to accumulate Calcein (panel **B**, gate 1). The behaviour of these cells remained stable in subsequent experiments suggesting that they are not readily transfectable and that their lack of accumulation of Calcein in the presence of inhibitors is not due to endogenous ABCC1 expression (see below). These cells likely represent a stem-like population within the HEK293T culture which have not been investigated further but which have been excluded from the analysis. In the absence of an antibody recognising an extracellular epitope of ABCC1, it was not possible to correlate definitively the transporter expression directly with reduced accumulation of Calcein. However, by co-transfection of pDsRed, encoding red-fluorescent protein, into the transient-transfection mix with pcDNA3-ABCC1 (in a 1:3 w/w ratio, respectively) transfected cells could be identified by their red fluorescence in flow cytometry (panel **C**, gate 3). Addition of Calcein-AM to the cells showed that red-fluorescent transfected cells had reduced Calcein accumulation (panel **C**,

gate 3 and red histogram in panel **E**) compared to the untransfected population (panel **C**, gate 2 and blue histogram in panels **E**). It is therefore very likely that the red-fluorescent cells also took up pcDNA3-ABCC1 and the lowered Calcein content is due to the active efflux of Calcein-AM (and/or Calcein) by the ABCC1 transporter. The level of efflux activity was then quantified by calculating the fold-difference in Calcein accumulation between the untransfected cells and the red-fluorescent population. This provided a reproducible measure of ABCC1 activity that could be used to characterise putative inhibitors. The Calcein content of the untransfected cells and red-fluorescent transfected cells could be modulated by the addition of inhibitor (S3 500 μM in the example shown in panel **D** and the red histogram in panel **F**). Titration of the drug concentration in the experiment allowed the efficacy and potency of S3 to be compared with its parent drug Sulindac.

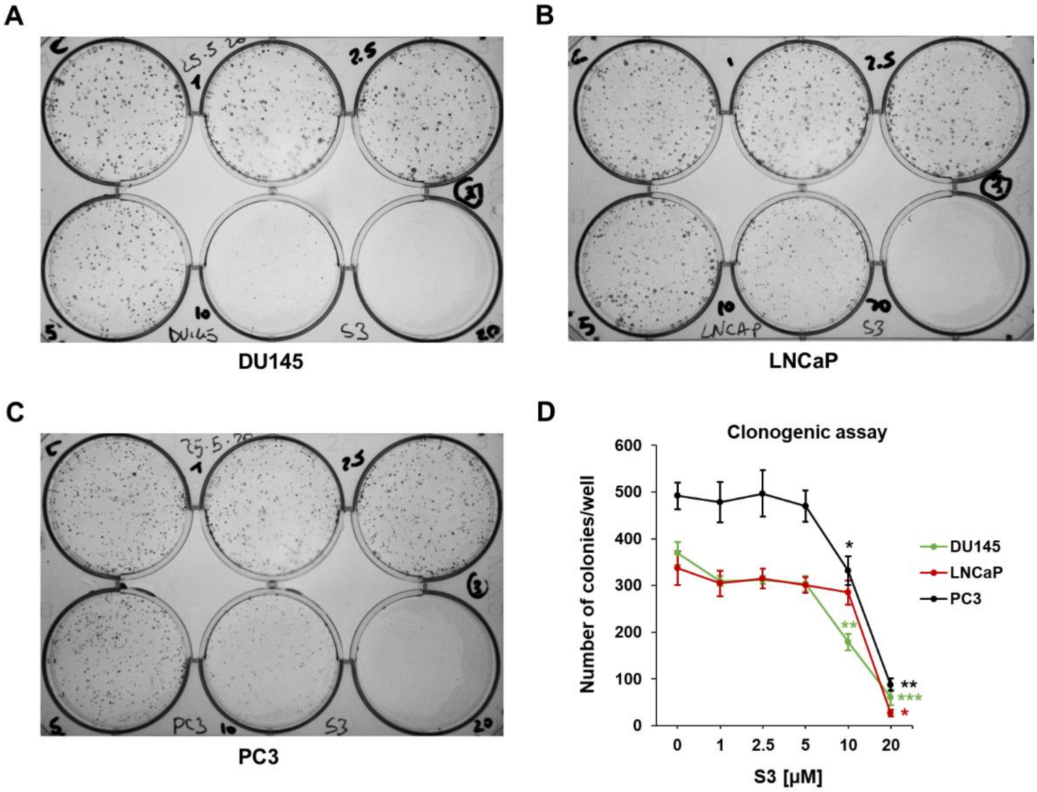

Figure S7. S3 reduces the ability of prostate cancer cells to form colonies in clonogenic assays. DU145 (**A**,**D**), LNCaP (**B**,**D**) and PC3 (**C**,**D**) cells were plated as single cells in 6 well plates and incubated for 10 days in media supplemented with the indicated concentrations of S3. Control cells were incubated with vehicle alone. (**A–C**) Representative images of 6 well plates. (**D**) Data indicate number of colonies/well and are means ± SEM of $n = 3$ independent experiments. * $p < 0.05$, ** $p < 0.01$, *** $p < 0.001$ vs results from corresponding control cells.

A

Dose S3 [µM]	Dose Docetaxel [nM]	Effect	CI
2.5	2.0	0.09823	0.25580
2.5	5.0	0.16651	0.38111
2.5	10.0	0.29572	0.50641
5.0	1.0	0.04094	0.56349
5.0	2.0	0.13396	0.25637
5.0	5.0	0.15794	0.43526
5.0	10.0	0.31870	0.49624

B

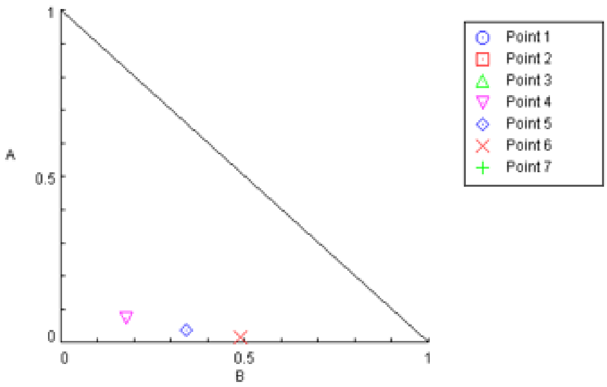

C

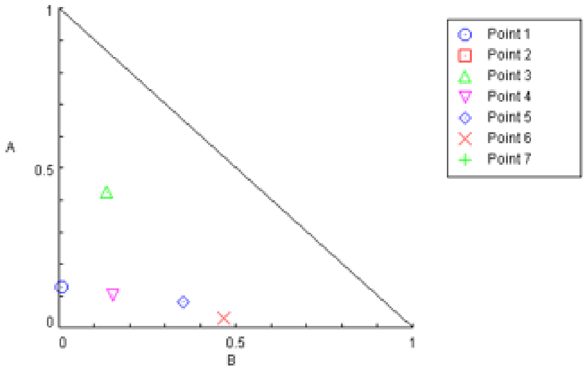

Figure S8. CompuSyn Report—PC3 cells. PC3 cells were treated with the indicated concentrations of S3 and Docetaxel. Viability was assessed by MTT assays after 72 h. (**A**) Table displays combination index (CI) data for the indicated drug combinations. (**B**,**C**) Normalized isobologram for combinations of Docetaxel with 2.5 µM S3 (**B**) or with 5 µM S3 (**C**).

A

Dose S3 [µM]	Dose Docetaxel [nM]	Effect	CI
2.5	2.0	0.26499	0.33832
2.5	5.0	0.40194	0.47152
2.5	10.0	0.51757	0.67975
5.0	1.0	0.04707	0.95502
5.0	2.0	0.39739	0.38273
5.0	5.0	0.48788	0.51050
5.0	10.0	0.59734	0.67940

B

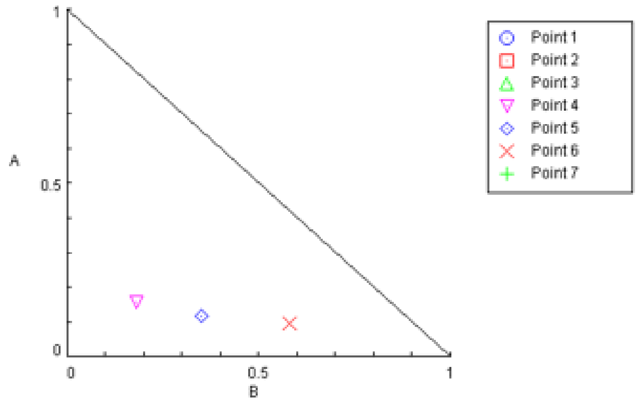

C

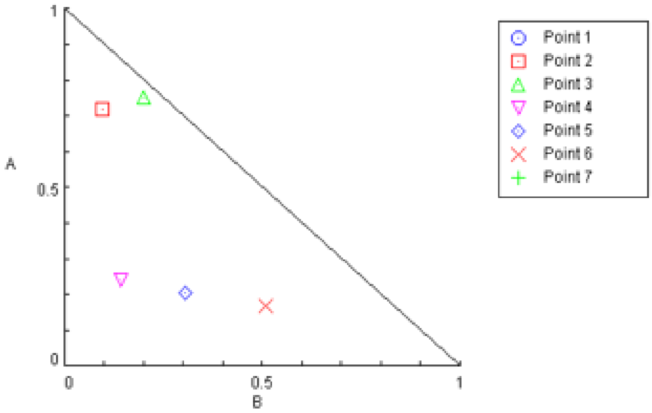

Figure S9. CompuSyn Report—LNCaP cells. LNCaP cells were treated with the indicated concentrations of S3 and Docetaxel. Viability was assessed by MTT assays after 72 h. (**A**) Table displays combination index (CI) data for the indicated drug combinations. (**B**,**C**) Normalized isobologram for combinations of Docetaxel with 2.5 µM S3 (**B**) or with 5 µM S3 (**C**).

A

Dose S3 [µM]	Dose Docetaxel [nM]	Effect	CI
2.5	2.0	0.08778	0.55673
2.5	5.0	0.27133	0.59053
2.5	10.0	0.66474	0.52032
5.0	1.0	0.19216	0.58243
5.0	2.0	0.19027	0.67286
5.0	5.0	0.43497	0.65615
5.0	10.0	0.58020	0.76335

B

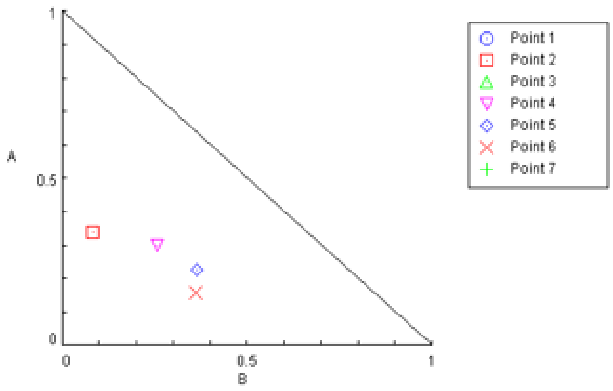

C

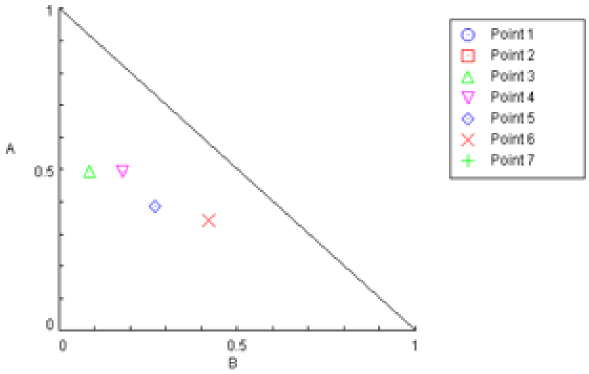

Figure S10. CompuSyn Report—DU145 cells. DU145 cells were treated with the indicated concentrations of S3 and Docetaxel. Viability was assessed by MTT assays after 72 h. (**A**) Table displays combination index (CI) data for the indicated drug combinations. (**B**,**C**) Normalized isobologram for combinations of Docetaxel with 2.5 µM S3 (**B**) or with 5 µM S3 (**C**).

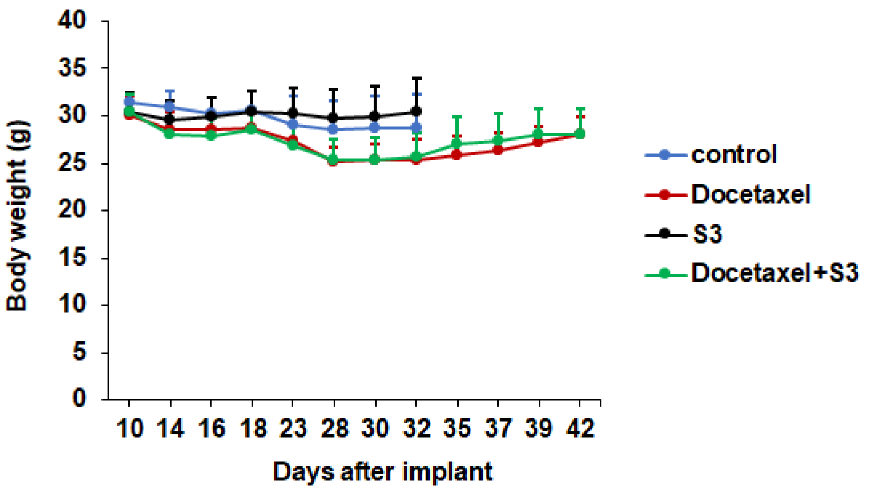

Figure S11. Body weights of mice used in experiment described in Figure 7B.

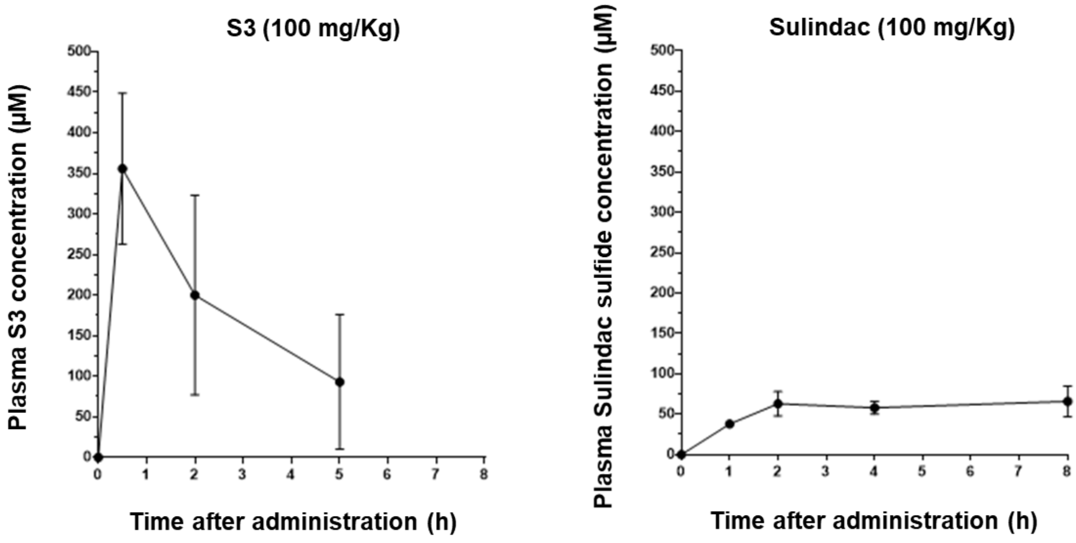

Figure S12. Plasma pharmacokinetics of S3 in C57BL/6N mice (left panel) and Sulindac in athymic nude mice (right panel) after a single oral administration of a 100 mg/kg dose. Mean ± SD (n = 3).

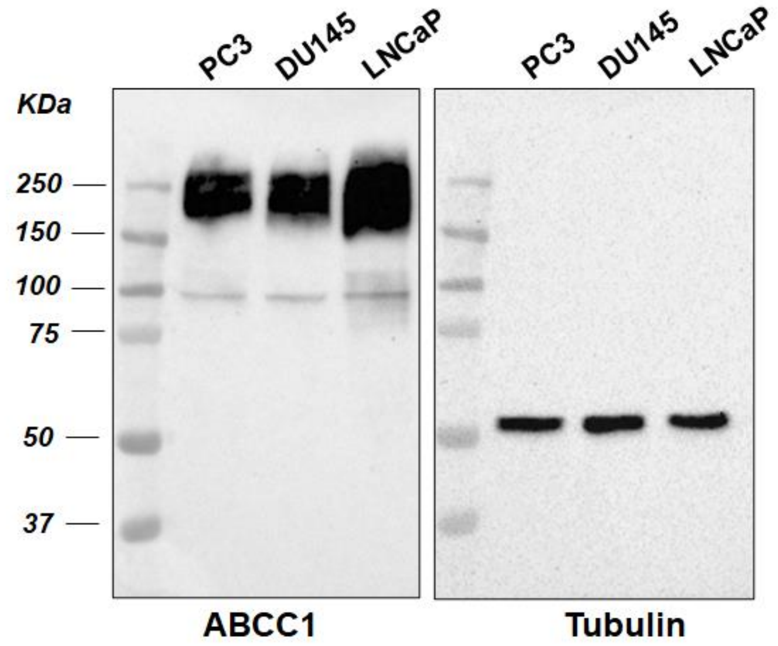

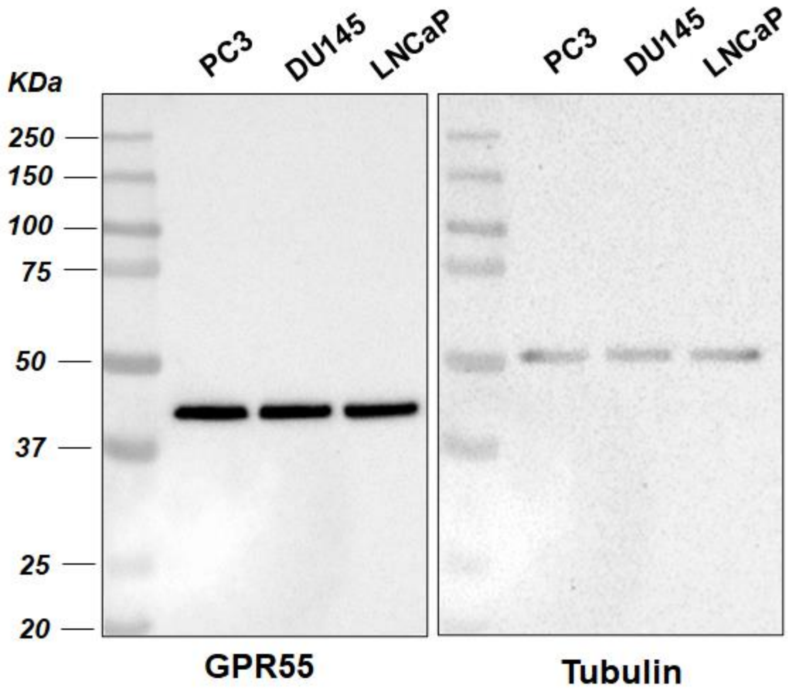

Figure S13. Whole blots of images shown in Figure 1. Expression of ABCC1 and GPR55 was assessed by Western blotting analysis of lysates from the indicated prostate cancer cell lines. Membranes were stripped and re-incubated with anti-Tubulin to confirm equal loading.
